# Comparison of Statistical Data Models for Identifying Differentially Expressed Genes Using a Generalized Likelihood Ratio Test

**DOI:** 10.4137/grsb.s381

**Published:** 2008-03-17

**Authors:** Kok-Yong Seng, Robb W. Glenny, David K. Madtes, Mary E. Spilker, Paolo Vicini, Sina A. Gharib

**Affiliations:** 1 Department of Bioengineering, University of Washington, Seattle, Washington, U.S.A; 2 Department of Medicine, University of Washington, Seattle, Washington, U.S.A; 3 Department of Physiology and Biophysics, University of Washington, Seattle, Washington, U.S.A; 5 Fred Hutchinson Cancer Research Center, Seattle, Washington, U.S.A

**Keywords:** microarray data analysis, generalized likelihood ratio test, statistical error model, parametric t-test, receiver operating characteristic curve

## Abstract

Currently, statistical techniques for analysis of microarray-generated data sets have deficiencies due to limited understanding of errors inherent in the data. A generalized likelihood ratio (GLR) test based on an error model has been recently proposed to identify differentially expressed genes from microarray experiments. However, the use of different error structures under the GLR test has not been evaluated, nor has this method been compared to commonly used statistical tests such as the parametric *t*-test. The concomitant effects of varying data signal-to-noise ratio and replication number on the performance of statistical tests also remain largely unexplored. In this study, we compared the effects of different underlying statistical error structures on the GLR test’s power in identifying differentially expressed genes in microarray data. We evaluated such variants of the GLR test as well as the one sample *t*-test based on simulated data by means of receiver operating characteristic (ROC) curves. Further, we used bootstrapping of ROC curves to assess statistical significance of differences between the areas under the curves. Our results showed that i) the GLR tests outperformed the *t*-test for detecting differential gene expression, ii) the identity of the underlying error structure was important in determining the GLR tests’ performance, and iii) signal-to-noise ratio was a more important contributor than sample replication in identifying statistically significant differential gene expression.

## Introduction

The development of microarray technology has been phenomenal in the past decade, with transcriptional profiling now a standard tool in many genomics research laboratories (Ginsberg and Mirnics, 2006; Rosa et al. 2006). The rapid development and acceptance of this method can be attributed to the fact that microarrays permit the simultaneous measurement of thousands of gene expressions on a single platform instead of analyzing them on a gene-by-gene basis. One major application of this technology is the identification of genes that are differentially expressed across various experimental conditions. Such differentially expressed genes may be implicated in biological pathways of interest and can help our understanding of disease mechanisms and treatment strategies (Phan et al. 2006; Cowell and Hawthorn, 2007).

A popular method to detect difference in gene expression has been the use of fold-change cutoffs (Chattopadhyay et al. 2007; Shimada et al. 2007). This approach seeks genes whose expression intensities change, for example, by a factor of two or more between control and treatment samples. However, the fixed threshold cutoff method is not based on specific data modeling assumptions and is statistically inefficient because it cannot account for the numerous systemic and biological variations inherent in a microarray experiment (Jaluria et al. 2007). Another commonly used method is the traditional parametric *t*-test. The performance of the *t*-test depends on the sample size, and whether the expression intensities can be assumed as normally distributed (Riva et al. 2005).

To address the need for a better statistical framework for microarray data analysis, various investigators have proposed the quantification of measurement errors associated with gene expression intensities (Ideker et al. 2000; Tu et al. 2002). In particular, parameters of a statistical data model, which account for potential error sources, can be estimated using the maximum-likelihood estimation (MLE) method. A generalized likelihood ratio (GLR) test can then be applied to identify genes whose expression levels are statistically different. A crucial step in the GLR test lies in the selection of the underlying error structure summarizing the influence of multiple sources of variation in microarray studies. Several models have been proposed for measurement errors in microarray data (Ideker et al. 2000; Rocke and Durbin, 2001; Tu et al. 2002). All of these models account for the observation that the variance of expression data of a gene increases with its mean. Ideker et al. have shown that a model that reflects two types of error, one additive and one multiplicative, can adequately model microarray data at varying intensity levels (Ideker et al. 2000). The multiplicative error term accounts for intraarray variance that influences individual study parameter estimates. The additive error component captures the influence of interarray variations during replicate experiments. This GLR model has been applied to non-logarithm transformed intensity levels from cDNA microarray experiments.

Although various other implementations of error structures under the GLR test have been presented, no systematic comparative studies of their performance have been published. It has been reported that logarithm transformation can improve the normality of expression levels and help equalize variance, since raw intensities follow a lognormal distribution (Baldi and Long, 2001; Quackenbush, 2002). To the best of our knowledge, no statistical data model expressed in the log scale has been implemented in the GLR test. Furthermore, it is unclear how the GLR method compares to a traditional statistical test such as the parametric *t*-test in detecting differential gene expression. Our primary aim of this paper was to assess the performance of several variants of the GLR test—with error models fitting raw and log-transformed expression intensities—and the one sample *t*-test using simulated data derived from actual cDNA microarray experiments. Our null hypothesis for the implemented tests was that each gene was not differentially expressed under control and experimental conditions. When identifying differentially expressed genes based on the expression levels of thousands of genes, however, statistical difficulties can arise due to massive multiple hypothesis testing, where the finite significance of each test produces many false positives overall (Norris and Kahn, 2006). Though the issue of multiple testing is crucial and warrants reliable correction techniques, we have restricted the scope of our present work to comparing tests for assessing marginal (i.e. per gene) significance without multiplicity adjustment. We compared the power of these statistical tests by receiver operating characteristic (ROC) curves (Hanley and McNeil, 1982). Specifically, we determined the ROC summary index (area under ROC curve) and its confidence interval using the bootstrap technique (Efron and Gong, 1983; Efron and Tibshirani, 1986). A secondary objective of the present study was to investigate the relative importance of signal-to-noise ratio and replication in the determination of differential gene expression.

## Methods

### Experimental study

Gene expression data were generated from lung tissues of mice exposed to chronic hypoxia. During chronic hypoxia, mice develop pulmonary hypertension and pulmonary vascular remodeling (Gharib et al. 2005). However, this *in vivo* perturbation leads to only a modest degree of differential gene expression with relatively few genes changing by more than two-fold (Gharib et al. 2005). In contrast, a typical yeast experiment such as response to galactose stimulation results in a much more profound differential gene expression profile (Ideker et al. 2000).

Four, 8 week old, male Balb/C mice (The Jackson Laboratory, Bar Harbor, ME) were exposed to 21 days of hypobaric hypoxia (0.5 atm). Four control mice were housed at sea level for the duration of the experiment (normoxia). On day 21, all mice were sacrificed, whole lungs removed, total RNA isolated and hybridized to cDNA microarrays consisting of 5313 murine genes and expressed sequence tags. The RNA from the four control mice was pooled and used as reference for all microarray experiments. Four microarray replications with dye swapping were performed for three of the hypoxic mice; the fourth hypoxic mouse was studied in triplicate for a total of 15 labeling experiments. By performing replications for each animal and using multiple animals, our experiment design captured both the biological and technical noise in differential gene expression during hypoxic exposure.

### Data simulation

The first step in our data simulation strategy was to construct a realistic statistical model based on the experimental microarray data, and then to use this model to generate artificial gene expression values with varying statistical characteristics. Note that our statistical model described data from a cDNA microarray experiment in which control and treatment samples are hybridized on the same array. Based on exploratory data analysis of our 15 replicate microarray experiments, we observed that the variability of each gene’s intensity under hypoxic or control conditions in the log scale was approximately Gaussian. In addition, we also noted that two intensity measurements (under hypoxia and normoxia) of the same spot were highly correlated. These observations were in agreement with published findings (Ideker et al. 2000; Baldi and Long, 2001; Rocke and Durbin, 2001; Tu et al. 2002).

We simulated 2000 genes in this study: 1000 genes were defined as differentially expressed (*diffgenes*) and 1000 genes as unchanged (*nullgenes*) during hypoxia relative to the unperturbed normoxic condition. Each *diffgene* had a unique expected value under hypoxic and normoxic conditions, in addition to a noise-induced error per replication. By definition, each *nullgene* had the same expected mean intensity during hypoxia and normoxia but different measurable values from one replication to another due to experimental variability. We used the following model for simulating the observed paired intensities, (*x, y*), of background-subtracted and normalized *diffgenes* and *nullgenes* during replicate experiments:

(1a) $$\begin{array}{l} {\text{log}}_{2}{\left[x_{diffgene}\right]}_{n_{ik}}=\mu_{diffgene_{n_{i}}}+{ɛ}_{n_{ik}}\\ \text{for the control }(\text{normoxia})\;\text{sample}\\ {\text{log}}_{2}{\left[y_{diffgene}\right]}_{h_{ik}}=\mu_{diffgene_{h_{i}}}+{ɛ}_{h_{ik}}\\ \text{for the treatment }(\text{hypoxia})\;\text{sample} \end{array}$$

(1b) $$\begin{array}{l} {\text{log}}_{2}{\left[x_{nullgene}\right]}_{n_{jk}}=\mu_{nullgene_{n_{j}}}+{ɛ}_{n_{jk}}\\ \text{for the control }(\text{normoxia})\;\text{sample}\\ {\text{log}}_{2}{\left[y_{nullgene}\right]}_{h_{jk}}=\mu_{nullgene_{h_{j}}}+{ɛ}_{h_{jk}}\\ \text{for the treatment }(\text{hypoxia})\;\text{sample} \end{array}$$

for *diffgenes i = 1,2,…,N*, *nullgenes j = 1,2,…,M*, and replications *k = 1,2,…,r*. Subscript *h* refers to hypoxia and *n* to normoxia; *μ_diffgene_h__* (or *μ_nullgene_h__*) is the expected “true” intensity under hypoxia and *μ_diffgene_n__* (or *μ_nullgene_n__*) is the expected “true” intensity during normoxia (in the log scale). Recall that *μ_diffgene_n_i___* ≠ *μ_diffgene_h_i___* and *μ_nullgene_n_j___* = *μ_nullgene_h_j___*. For each gene, the random error terms during hypoxia and normoxia, *ɛ_h_* and *ɛ_n_*, were chosen from an independent bivariate normal distribution with mean zero, variance under hypoxia (*ω_h_*) and normoxia (*ω_n_*), and correlation (*ρ_h,n_*).

In order to create realistic data simulations, we chose the parameters for the above model from our set of 15 replicate microarray experiments. We randomly selected 1000 genes from this data set and assigned their mean expression values during hypoxia and normoxia as our 1000 simulated *μ_diffgene_h__* and *μ_diffgene_n__* (1a). In addition, we used the variance of each of these 1000 genes over 15 replicate experiments as the variance of the error component of each assigned *diffgene* (the same procedure applied for each *nullgene*). For the *nullgenes*, we chose a different set of 1000 genes from the original data set, and assigned, by definition, *μ_nullgene_h__* = *μ_nullgene_n__* (1b). The correlation term for the bivariate normal distribution for each *diffgene* and *nullgene* was obtained directly from the expression measurements in these experiments. Note that there was a range of differential gene expression values among the *diffgenes* since each gene had a different *μ* (during hypoxia and normoxia) and each replicate had a different *ɛ*. Similarly, although each *nullgene* was defined to have equal mean intensity during hypoxia and normoxia, each replication resulted in a different expression value because of the error term *ɛ*. Gene expression values were now derived by randomly drawing error terms from the bivariate Gaussian distribution unique to each of the 2000 genes. Simulated gene expression replicates based on this approach should capture both the biological variability and the technical “noise” of the original microarray experiments. Furthermore, since these simulated log-transformed intensities were normally distributed, parametric approaches such as the parametric *t*-test and GLR test could be applied to identify differentially expressed genes.

We denoted data sets with s = 1, 2 or 3 as data containing ‘low’, ‘medium’ or ‘high’ signal-to-noise ratios respectively. We generated the ‘low’ signal-to-noise ratio (s = 1) data based on differential gene expression values in our experimental model of hypoxic pulmonary hypertension. As discussed above, we believe this perturbation resulted in only a modest level of differential gene expression. Therefore, we increased the difference between *μ_diffgene_h__* and *μ_diffgene_n__* for the 1000 *diffgenes* by two-fold to obtain ‘medium’ (s = 2) and three-fold to obtain ‘high’ (s = 3) signal-to-noise ratios. These data sets were aimed at modeling data generated during more profound perturbations such as ionizing radiation or yeast sporulation studies. Similarly, replications with r = 2, 3 or 4 and 6 or 8 were data sets containing ‘low’, ‘medium’ or ‘high’ number of replications per gene, respectively. In total, 15 sets of simulated data at each signal-to-noise ratio (s = 1, 2 and 3) and replication number (r = 2, 3, 4, 6 and 8) were generated. These data sets therefore differed in terms of quality, with r2s1 (2 replications per gene and a signal-to-noise ratio of 1) having the lowest and r8s3 (8 replications per gene and a signal-to-noise ratio of 3) having the highest quality respectively. This pool of 15 data sets constituted this study’s original simulated sample. Because we knew *a priori* which genes were differentially expressed and which were not, altering signal-to-noise ratios and replications per gene allowed us to compare the robustness and performance of various statistical tests in detecting differentially expressed genes.

### Statistical data models

We implemented the statistical data model proposed by Ideker and co-authors (Ideker et al. 2000). In addition, motivated by empirical observations, we also devised three additional error models that considered log-transformed expression intensities—termed GLR1, GLR2 and GLR3—for analysis using the GLR test. These three error structures have the following generic form:

(2) $$\begin{array}{l} {\text{log}}_{2}{\left[x\right]}_{ij}=\mu_{x_{i}}+f(\mu_{x_{i}}){ɛ}_{x_{ij}}^{H}+{ɛ}_{x_{ij}}^{A}\\ \text{for the control sample},\text{and}\\ {\text{log}}_{2}{\left[y\right]}_{ij}=\mu_{y_{i}}+f(\mu_{y_{i}}){ɛ}_{y_{ij}}^{H}+{ɛ}_{y_{ij}}^{A}\\ \text{for the treatment sample} \end{array}$$

where (*μ_x_i__*, *μ_y_i__*) is the pair of mean intensities for gene *i*, multiplicative error *ɛ^H^* is drawn from a bivariate normal distribution with mean 0, variances *ω_x_^H^* and *ω_y_^H^*, and correlation 0 (i.e.*ɛ*^H^ ~ BN(0 0, *ω_x_^H^*, *ω_y_^A^*, *ρ*)) (*ρ* denotes correlation. The correlation term models the observation that the log-transformed expression intensities (under control and treatment conditions) per spot consistently correlate over repeated measurements. *ɛ^H^* and *ɛ^A^* are assumed to be independent of each other. Additionally, *f*(*μ*) allows one to incorporate into the statistical error structure the common observation that the standard deviation is dependent on the magnitude of intensity (mean) (Ideker et al. 2000; Baldi and Long, 2001; Rocke and Durbin, 2001; Tu et al. 2002).

For the simplest case, we set *f* (*μ_x_i__*) and *f* (*μ_y_i__*) equal to 1. Under this model, the measured expression intensity is dependent on a linear combination of normal error components. Hence, GLR1 is:

(3) $$\begin{array}{l} {\text{log}}_{2}{\left[x\right]}_{ij}=\mu_{x_{i}}+{ɛ}_{x_{ij}}^{H}+{ɛ}_{x_{ij}}^{A}\\ {\text{log}}_{2}{\left[y\right]}_{ij}=\mu_{y_{i}}+{ɛ}_{y_{ij}}^{H}+{ɛ}_{y_{ij}}^{A} \end{array}\;\;\;\text{GLR}1$$

Based on (2), GLR2 was constructed by equating *f* (*μ_x_i__*) and *f* (*μ_y_i__*) to *μ_x_i__* and *μ_y_i__* respectively (4), so that the variance could be approximately proportional to the mean intensity (Ideker et al. 2000; Tu et al. 2002). In GLR3, *f* (*μ_x_i__*) = 1/*μ_x_i__* and *f* (*μ_y_i__*) =1/*μ_y_i__*. In GLR3, we explicitly modeled the observation that correlation between expression levels under control and treatment conditions decreases at lower intensities by weighting the multiplicative error term using the reciprocals of the mean intensity, as shown in (5).

(4) $$\begin{array}{l} {\text{log}}_{2}{\left[x\right]}_{ij}=\mu_{x_{i}}+\mu_{x_{i}}{ɛ}_{x_{ij}}^{H}+{ɛ}_{x_{ij}}^{A}\\ {\text{log}}_{2}{\left[y\right]}_{ij}=\mu_{y_{i}}+\mu_{y_{i}}{ɛ}_{y_{ij}}^{H}+{ɛ}_{y_{ij}}^{A} \end{array}\;\;\;\text{GLR}2$$

(5) $$\begin{array}{l} {\text{log}}_{2}{\left[x\right]}_{ij}=\mu_{x_{i}}+\frac{1}{\mu_{x_{i}}}{ɛ}_{x_{ij}}^{H}+{ɛ}_{x_{ij}}^{A}\\ {\text{log}}_{2}{\left[y\right]}_{ij}=\mu_{y_{i}}+\frac{1}{\mu_{y_{i}}}{ɛ}_{y_{ij}}^{H}+{ɛ}_{y_{ij}}^{A} \end{array}\;\;\;\text{GLR}3$$

Describing raw intensity data, the error structure proposed by Ideker et al. (2000) is:

(6) $$\begin{array}{l} x_{ij}=\mu_{x_{i}}+\mu_{x_{i}}{ɛ}_{x_{ij}}^{H}+{ɛ}_{x_{ij}}^{A}\\ y_{ij}=\mu_{y_{i}}+\mu_{y_{i}}{ɛ}_{y_{ij}}^{H}+{ɛ}_{y_{ij}}^{A} \end{array}\;\;\;\text{VS}$$

(6) was implemented in a public-domain computer program, Vera and Sam (http://db.systemsbiology.net/software/VERAandSAM. ) (Ideker et al. 2000). Henceforth, we refer to this statistical error model as VS.

Although GLR1—3 may appear similar to the data simulation model in (1), there were fundamental differences between them. Crucially, we used a different Gaussian distribution for each of the 2000 genes in our data simulation while the GLR tests were applied to each gene to determine whether, under a single statistical data model encompassing all 2000 genes, the expression levels under control and experimental conditions were different. For example, although the GLR1 structure (3) may appear very similar to the data simulation model (1), a different Gaussian distribution was sampled for each of the simulated genes, whereas the GLR test was performed for each gene to assess whether applying one distribution to the entire set of 2000 genes can discriminate expression levels between normoxic and hypoxic states. Furthermore, since the parametric *t*-test was applied to each gene separately without the requirement of a constant variance or a constant coefficient of variation across genes, we expected the *t*-test to be well suited in detecting differential gene expression.

### GLR method

Details of the GLR test can be found in Ideker et al. (2000). For illustrative purposes, we will briefly discuss the GLR test for VS as defined by (6). This statistical error model is dependent on five gene-independent parameters ***β*** = (*ω_x_^H^*, *ω_y_^H^*, *ω_x_^A^*, *ω_y_^A^*, *ρ*) as well as a mean pair per gene ***μ*** = [(*μ_x_1__*, *μ_y_1__*), …, (*μ_x_N__*, *μ_y_N__*)]. The probability density function for gene *i* is *P = P (x_ij_, y_ij_*|***β***, (*μ_x_i__*, *μ_y_i__*) The GLR method is essentially composed of two consecutive steps: parameter estimation followed by likelihood ratio test. In the Vera (Variability and ERror Assessment) program, ***β*** and *μ* are first estimated from the expression levels of all genes via MLE. MLE is the procedure of finding the value of one or more parameters for a given statistic that makes the known likelihood distribution a maximum (Kendall and Stuart, 1979). Likelihood functions, for gene *i* and over all genes, are defined as, respectively:

(7a) $$L_{i}\left(\beta ,\mu_{x_{i}},\mu_{y_{i}}\right)=\prod_{j=1}^{M}P\left(x_{ij},y_{ij}|\beta ,\mu_{x_{i}},\mu_{y_{i}}\right)$$

(7b) $$L_{i}\left(\beta ,\mu \right)=\prod_{i=1}^{N}L_{i}\left(\beta ,\mu_{x_{i}},\mu_{y_{i}}\right)$$

The MLE parameter values that maximize *L*, denoted by ***β̂*** and ***μ̂***, represent the estimates for the true parameters of the error model. In this study, we modified the original Vera program to incorporate the new statistical data models, namely GLR1, GLR2 and GLR3. All stages of the optimization were performed in C code (<10 min on a Pentium 4 computer with 1.4 GHz, 768 MB DDRAM memory for a data set containing 2000 genes per microarray and 4 replications per gene). All parameter values converged after 100–200 iterations.

To determine whether, under the pre-specified error model, individual genes are significantly differentially expressed (i.e. (*μ_x_i__* ≠ (*μ_y_i__*) between the two cell populations, a likelihood ratio test is subsequently performed, which assumes a univariate normal distribution for the expression data of a gene not differentially expressed (null hypothesis) and a mixture of two univariate normal distributions (with different means) for the expression data of a gene differentially expressed under control and treatment conditions (alternative hypothesis). To this end, the GLR test statistic *λ_i_* is computed using SAM (Significance of Array Measurement) (Kendall and Stuart, 1979):

(8) $$\lambda_{i}=-2\times \text{ln}\left(\frac{{\text{max}}_{\mu }L_{i}(\beta ,\mu ,\mu )}{{\text{max}}_{\mu_{x},\mu_{y}}L_{i}(\beta ,\mu_{x},\mu_{y})}\right)$$

Two maximizations are performed in (8): in the numerator, the constraint *μ_x_* = *μ_y_* = *μ* is imposed (null hypothesis), whereas in the denominator, the optimization is unconstrained, i.e. *μ_x_* ≠ *μ_y_* (alternative hypothesis). In the case where *μ_xi_* = *μ_yi_*, *λ_i_* follows a *χ*^2^ distribution with one degree of freedom. To select differentially expressed genes, a critical cutoff value *λ_c_* must be determined beforehand based on the results of a set of control to control microarray experiments. However, since our data simulation provides us *a priori* information on which genes are differentially expressed and which ones are unchanged we can circumvent the control to control experiments and use ROC curves to compare the performance of variants of the GLR test and the *t*-test.

### Parametric *t*-test

The *t*-test statistic and its variants are commonly used to detect differential gene expression because genes deemed as significantly expressed have maximal difference in mean expression values between two groups and minimal variation of expression within each group. In this study, we compared the log ratios of spot intensities between control and treatment groups using the parametric one-sample *t*-test (Skokal, 1995).

### Performance of statistical tests on simulated microarray data

ROC curve analysis was used for performance evaluation. Expression status of each gene (i.e. whether a gene was classified as a *diffgene* or *nullgene*) was known and served as the reference or ‘gold standard’. The area under the ROC curve (AROC) was used as a unidimensional summary of the ability of the statistical test to discriminate between *diffgene* or *nullgene*, ranging from 0.5 for a test with no diagnostic capability to 1 for a test with perfect separation of the two groups. The ROC curve was derived by: 1) ranking values of the test metric (i.e. *t*-statistic or *λ*) of each gene; and 2) identifying the correctly and incorrectly assigned *diffgenes* and *nullgenes* by varying a threshold; and 3) computing the full range of true-positive fraction or sensitivity values, and the false-positive fraction or 1-specificity values. ROC curves thus provide a more complete measure of a test’s discriminatory ability than the choice of an arbitrary and isolated (sensitivity, specificity) point. Once the ROC curve was obtained, the area underneath it was computed to estimate AROC.

Furthermore, a parametric bootstrap procedure was used to quantify the precision of estimates of AROC and to approximate the probability of rejecting the null hypothesis when comparing the AROC’s of two different statistical tests. Bootstrapping provides a way of making probability-based, assumption-free inferences about a population characteristic (e.g. AROC) without strict distributional assumptions (Efron and Gong, 1983; Efron and Tibshirani, 1986). In short, one thousand samples of *diffgenes* and *nullgenes* of size 2000 each were generated by sampling with replacement from the original set of 2000 genes in each of the 15 original samples. The ROC curve and its associated AROC for each re-sample were then determined. The 95% confidence interval for AROC under each data condition and statistical test was derived from these bootstrap re-samples by finding the minimum interval [*I*_1_, *I*_2_] for which the probability *P*_AROC_(*I*_1_ =< AROC < *I*_2_) = 0.95. To compare the performance between two statistical methods, we computed a *z*-statistic using the bootstrap to generate the variance of the AROC estimates (Margolis et al. 2002):

(9) $$z=\frac{A_{1}-A_{2}}{\sqrt{{\left({SE}_{A_{1}}\right)}^{2}+{\left({SE}_{A_{2}}\right)}^{2}-Cov(A_{1},A_{2})}}$$

where *A*_1_ and *A*_2_ refer to the AROC’s from the two statistical tests to be compared, SE is the standard error, which was estimated using the bootstrap, and *Cov*(*A*_1_, *A*_2_) represents the covariance of *A*_1_ and *A*_2_. *Cov*(*A*_1_, *A*_2_) was derived by taking the product of the correlation between the bootstrapped samples (corresponding ROC curves will be correlated since they are derived based on the analysis of the same set of bootstrapped data) and standard deviations. Under the null hypothesis of no difference between two statistical tests, the *z*-statistic is approximately normally distributed (Margolis et al. 2002). In the present study, we chose a critical cutoff value of 1.96 to indicate that there was an approximately 5% chance (i.e. about 50 out of 1000 bootstrap re-samples) that any AROC difference could be attributed to pure chance and that the two ROC curves under comparison were in fact identical. Additionally, to determine if the predictive value of a statistical test was better than random chance, individual AROC was compared to 0.5 (corresponding to a coin toss) using a one-sided test at an *α* level of 5% (Delong et al. 1988).

## Results

### Overall performance of statistical tests on the original simulated data

**Figure 1:**
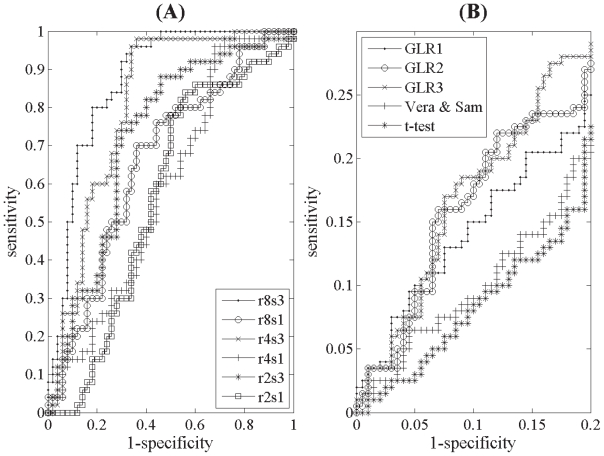
**(A)** ROC curves for different data conditions derived from the GLR test utilizing GLR3 as the underlying statistical data model. In the legend, r and s indicate replications per gene and signal-to-noise ratio of the data set condition respectively. The AROC for r8s3, r8s1, r4s3, r4s1, r2s3 and r2s1 were 0.862, 0.711, 0.847, 0.642, 0.806 and 0.604 respectively. **(B)** ROC curves for the r2s1 data set derived using variants of the GLR test and the *t*-test at high specificity range. Note that the ROC curves in **(A)** and **(B)** were generated based on the original simulated microarray data sample.

Representative ROC curves generated from our analysis are shown in Figure 1A. These curves were based on the GLR3 model using r2s1, r2s3, r4s1, r4s3, r8s1 and r8s3 original simulated data sets. The overall patterns of the ROC curves with respect to varying signal-to-noise ratio and number of replications per gene were similar among the statistical tests. As expected, the predictive power of the GLR tests and *t*-test increased with improvements in the sample size and/or signal-to-noise ratio of data sets. This indicates that the GLR tests and the *t*-test predicted more *diffgenes* correctly as the overall quality of microarray data improved. Also note that the curves corresponding to r4s3 (and even r2s3) were above those for r8s1, suggesting that stronger signals could lead to a higher test power than larger sample repeats. Individual AROC values ranged from 0.549 to 0.93, and one-sided test at an *α* level of 5% confirmed that the tests’ performance was always superior to random classification.

### Bootstrap comparison between GLR tests and *t*-test

**Figure 2:**
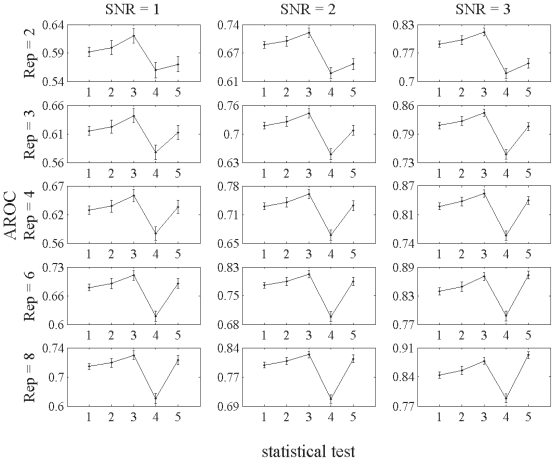
Mean (±S.D.) AROC’s out of 1000 bootstraps for the 5 statistical tests: 1. GLR1; 2. GLR2; 3. GLR3; 4. VS; and 5. *t*-test. Rep and SNR denote number of replications per gene and signal-to-noise ratio, respectively.

The mean (±S.D.) AROC’s calculated on the 1000 bootstrap replications for each of the five statistical tests and for each data condition are presented in Figure 2. The corresponding 95% confidence intervals of AROC’s are shown in Table 1. Table 2 shows the number of times out of 1000 that a statistical difference was detected between statistical tests for various data conditions using the bootstrap method and a *z*-statistic. For two statistical tests to be significantly different at the *P* < 0.05 level, it is expected that the null hypothesis would be rejected in 950 comparisons. These tables and figure reveal that, overall, the best discriminator was obtained by the GLR test employing GLR3 as its underlying statistical model, which attained significance with respect to all other tests in virtually every condition studied. There were significant differences between the GLR test employing the VS model and every other statistical test, indicating that the former was the least discriminatory. Of note, the *t*-test underperformed relative to the GLR tests when data quality was poor (low signal-to-noise or replication), but improved when the number of gene replicates and signal-to-noise were ‘high’ (r = 8, s = 3).

In Figure 1B, using the r2s1 data set, we explored an important aspect of the ROC curve to further emphasize differences among the tests: the trade-off between sensitivity and specificity. Here, we examined the sensitivity of the statistical tests at a high range of specificity since this situation is often of interest to biologists. Using ROC curves determined from the rest of the data sets, which we have not displayed for the sake of clarity, we concluded that the GLR3 error structure consistently achieved the highest level of sensitivity in the high specificity range.

### Effect of signal-to-noise ratio and replication number on test power

**Figure 3:**
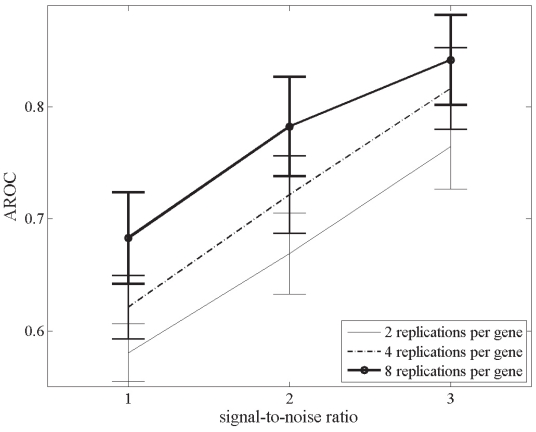
Mean (±S.D.) AROC’s out of 1000 bootstraps, sorted by signal-to-noise ratio of data sets. Note that for the largest number of gene replications considered in this study, the improvement in the tests’ performance from processing data sets with signal-to-noise ratio of ‘medium’ to processing data sets with signal-to-noise ratio of ‘high’ is reduced. For clarity’s sake, we have omitted the bootstrapped results from 3 and 6 replications per gene.

**Figure 4:**
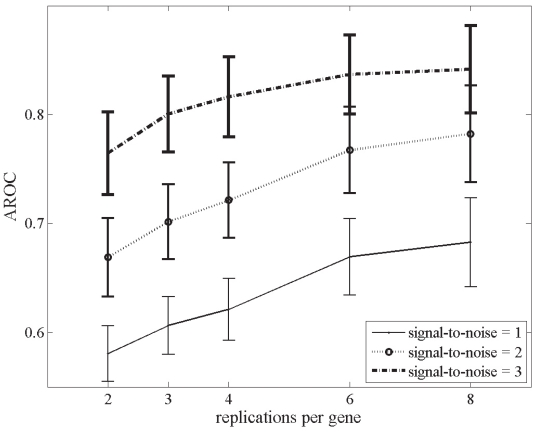
Mean (±S.D.) AROC’s out of 1000 bootstraps, sorted by number of replications per gene of data sets. Note that the AROC’s for ‘high’ signal quality data sets (signal-to-noise ratio of 3) with 3–8 replications per gene are comparable.

**Figure 5:**
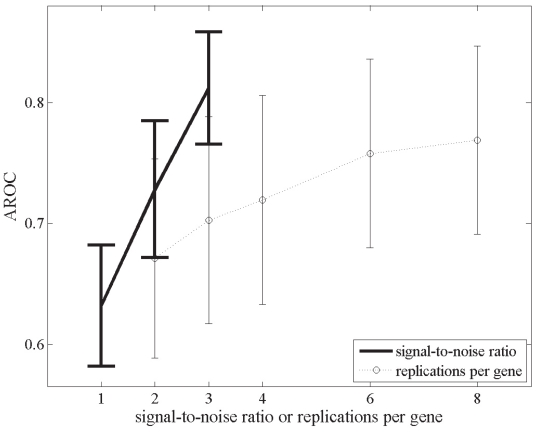
Mean (±S.D.) AROC’s out of 1000 bootstraps, sorted by signal-to-noise ratio or number of replications per gene of data sets.

The overall effects of signal-to-noise ratio and number of replications per gene on the statistical tests’ power using the bootstrap technique are presented in Figures 3–5. Improvement in the signal-to-noise ratio and increase in the number of replications both led to enhanced power. (Fig. 3). However, while increased replication improved test performance, this effect diminished as the signal-to-noise ratio of the data set improved. This observation is highlighted in Figure 4, where differences in AROC’s between ‘high’-signal-quality data sets (signal-to-noise ratio = 3) with replications ranging from 3 to 8 are negligible. The overall accuracy of the GLR tests and *t*-test improved by 34.4% when analyzing data sets with a signal-to-noise ratio of 1 compared to 3 (mean AROC of 0.614 and 0.821 respectively). In contrast, after increasing the number of replications by 4 fold (from 2 to 8 repeats per gene), we observed a lower increase of 11.9% (67% and 76% accuracy for 2 and 8 replicates respectively) in the average discriminating power (Fig. 5). Cumulatively, these findings indicate that signal-to-noise ratio was a more important contributor than sample replication to the performance of the statistical tests in identifying differentially expressed genes.

## Discussion

### Log-transformed GLR methods are superior to the parametric *t*-test

We compared the power of the *t*-test with that of the GLR test since the *t*-statistic has been commonly used to determine differentially expressed genes in microarray studies (Baldi and Long, 2001; Riva et al. 2005). Furthermore, the one sample *t*-test performs a gene-by-gene analysis, computing the sample variance for each gene in the analysis. Hence, this approach does not require a constant variance or a constant coefficient of variation across genes. This is consistent with our data simulation model where the variances of expression values of each gene were drawn from a unique bivariate Gaussian distribution. As a consequence, we expected the *t*-test to perform optimally in our simulated data sets. The GLR method, on the other hand, hinges on the usage of a single error structure to quantify the variability present across all 2000 genes (prior to gene-specific likelihood ratio tests) and therefore dramatically simplifies the complexity of the simulated gene expression data. Yet, our results indicate that the discriminatory ability of the GLR test was still equivalent or more powerful than the *t*-test for data sets containing ‘low’ and ‘medium’ gene replication number. It follows that GLR test’s assumption of a single statistical data model to quantitate the error distribution and structure of pooled gene expression data does not necessarily compromise its performance at identifying differentially expressed genes.

While there was an improvement in the *t*-test’s performance on data sets containing ‘high’ gene replications and signal-to-noise ratios, we showed that the GLR test (employing GLR3) could still attain a higher power than the *t*-test. A fundamental problem with employing tests dependent on Gaussian likelihoods in microarray studies (under the Gaussian model, the *t*-test is a generalized likelihood test) is that replications are often few due to costs or availability of biological material. Though such shortcomings can be addressed by increasing sample size, the potential gain in the statistical power of the GLR and *t*-test may be markedly offset by increases in cost and effort (Baldi and Long, 2001). This paper demonstrates that the GLR test represents a better framework for achieving a higher predictive power at detecting gene expression differences, especially at low numbers of replicates per gene.

### Error structure affects predictive power of GLR test

Overall, our results suggest that GLR3 was the best performing error structure compared with the GLR1, GLR2 and VS models. The GLR test using VS as its underlying error model consistently generated the lowest AROC’s for all bootstrap re-samples. We attribute this to the fact that the VS model’s error structure considers raw, non-log-transformed gene expression levels. We contend that such an assumption can potentially limit this test since logarithm transformation can improve the normality of expression intensities (Baldi and Long, 2001; Quackenbush, 2002). Among the statistical data models that considered log-transformed expression levels, GLR1 was the least discriminatory, implying that weighting the multiplicative error term by intensity enables the GLR test to achieve a higher predictive power (recall that *f*(*μ*) = 1 in GLR1 whereas *f*(*μ*) = *μ and* 1/*μ* in GLR2 and GLR3, respectively). Our results showing that the GLR3 was the most powerful statistical test. We believe that GLR3 performed better because its model structure accounts for two common traits associated with microarray expression data: (i) log-transformed microarray expression data are normally distributed (we transformed data to a logarithmic scale of base 2); (ii) correlation between log(x_i_) and log(y_i_) increases at higher spot intensities (we weighted one of two error components with the reciprocal of the mean spot intensity of each gene); and (iii) for each gene i, log(x_ij_) and log(y_ij_) are correlated over repeated measurements j. This suggests the importance of adjusting for a decrease in correlation between expression values of control and treatment genes at low intensities.

### Signal-to-noise ratio contributes more to test performance than replication number

A novel finding of this work was to show the greater influence of signal-to-noise ratio compared to gene replication on test performance. This implies that experiments that maximize the signal-to-noise ratio can facilitate to a larger extent the identification of significant differences in gene expression than those with more sample repeats. To the best of our knowledge, this study represents the first attempt to directly compare the influence of replication and signal-to-noise ratio on the performance of statistical analysis of microarray data, although several reports have commented on the significance and implications of each factor separately (Lee et al. 2000; Li et al. 2001; Wildsmith et al. 2001; Rosa et al. 2006). The greater influence of signal-to-noise ratio may have important implications in experiment design. Although in practice it may be difficult to modulate signal-to-noise, there are several venues for improvement. The quality of RNA is crucial for successful microarray hybrization, and this can be confirmed using microfluidics-based platforms such as the Agilent Bioanalyzer. Several techniques can be used to minimize technical noise, and microarrays from the same print batch can be used to reduce microarray to microarray variability. Importantly, our results also imply that if a biological system does have a high signal-to-noise ratio, then only a few replications are necessary. This may substantially cut the cost of expression profiling experiments.

### How many replications per gene?

It has been previously reported that performing microarray studies without sufficient replicates can lead to poor sensitivity and reliability (Lee et al. 2000; Wildsmith et al. 2001). However, the question ‘how many repeats are enough?’ is shaped by many potentially confounding factors such as the type of array equipment, laboratory technique, and, most importantly, the quality of the samples. Our study provides a framework to decide on the replication number per gene based on the overall signal-to-noise ratio of microarray data. More specifically, we recommend repeating array experiments 6–8 times for data with ‘poor’ and ‘medium’ signal-to-noise ratios, since the discriminatory ability of our tests levels off for sample size 6 or greater. In other words, data containing 6–8 replications per gene represent the best compromise between inferior signal quality and false positive rates. On the other hand, for data sets with ‘high’ signals (signal-to-noise ratio = 3), we show that 3 replications have essentially the same effect as 4 or more. This implies potential savings in future microarray studies since replications can be significantly reduced for such experiments. Taken together, our observations reinforce the notion that a successful microarray project is dependent on all steps of the process being accurately and consistently performed to maximize the reliability and significance of results. Here, we show that consideration of steps upstream of data processing, such as deciding on the proper number of microarray replications and minimizing technical/biological noise, may be necessary to ensure experimental and analytic accuracy.

An issue that bears consideration is that, in a practical setting, it may be difficult to assess the signal-to-noise ratio of actual microarray data. Signal-to-noise ratio depends on many factors, including quality of tissues, sample size relative to the number of variables to be classified, experimental variation, and inherently variable original signal intensities (Semenza, 2000; Norris and Kahn, 2006). In this light, our classification of signal-to-noise ratio levels may not be fully generalizable. To address this issue, we suggest independently quantifying the technical and biological sources of variation by, for instance, performing control-to-control experiments. Although this approach will require additional resources, it will provide valuable information on signal to noise proportion in the biological system of interest, allowing the investigator to choose the optimal number of replications to compensate for inadequate signal characteristics based on our present findings. In turn, this can lead to potential savings in experimental cost and resources.

There are several limitations in this study. First of all, we were constrained by how realistically our data simulation models the entire variability of microarray experiments. Furthermore, our experiments and data simulations were performed using cDNA microarrays and therefore might not be applicable to single dye oligonucleotide arrays such as the Affymetrix platform. We restricted our analyses to the GLR method and the parametric *t*-test. Additionally, the four statistical data models that were implemented under the GLR test do not represent the full spectrum of possible error structures. We also did not use partial AROC as a performance indicator to differentiate between statistical tests’ power at low false positive rate (or high specificity range). Finally, the problem of multiple comparisons was not addressed in this work because we were primarily interested in comparing the inherent statistical power of the *t*-test and GLR methods.

## Conclusions

In summary, although the present study depended on a specific set of simulated data derived from cDNA experiments and only the GLR test and the *t*-test statistical approaches were compared, our findings have three important implications for analysis of microarray data:

- The GLR method is more powerful than the parametric *t*-test for detecting differential gene expression. This underlines the importance of incorporating and modeling the error structure of microarray data during the development of future statistical tests.
- Within the GLR approach, an error structure that contains a multiplicative error term weighted by the reciprocal of mean expression intensity outperforms other models (GLR3).
- Designing experiments that maximize signal-to-noise ratio, instead of just raising the number of replicates per gene, can better identify differentially expressed genes in microarray data.

## Figures and Tables

**Table 1:** Bootstrap means and 95% confidence intervals of AROC’s for each data condition and each statistical test. GLR1, GLR2, GLR3 and VS refer to statistical data models that were implemented under the GLR test. Numbers that are highlighted in bold denote the highest scoring test per simulation condition.

Data condition	Mean AROC (95% confidence interval)				
	GLR1	GLR2	GLR3	VS	*t*-test
r8s3	0.82 (0.79–0.84)	0.84 (0.81–0.87)	0.87 (0.86–0.89)	0.79 (0.77–0.81)	0.89 (0.87–0.90)
r8s2	0.77 (0.75–0.80)	0.79 (0.77–0.82)	**0.82** (0.81–0.84)	0.71 (0.68–0.73)	0.81 (0.79–0.83)
r8s1	0.68 (0.65–0.70)	0.69 (0.67–0.72)	**0.72** (0.70–0.74)	0.61 (0.59–0.64)	0.71 (0.69–0.73)
r6s3	0.82 (0.79–0.84)	0.84 (0.81–0.86)	**0.87** (0.86–0.89)	0.79 (0.77–0.81)	0.87 (0.86–0.89)
r6s2	0.76 (0.73–0.78)	0.78 (0.75–0.81)	**0.81** (0.79–0.83)	0.70 (0.68–0.72)	0.79 (0.77–0.81)
r6s1	0.66 (0.64–0.68)	0.68 (0.65–0.71)	**0.71** (0.68–0.73)	0.61 (0.59–0.64)	0.69 (0.66–0.71)
r4s3	0.80 (0.78–0.83)	0.83 (0.80–0.85)	**0.85** (0.84–0.87)	0.76 (0.74–0.78)	0.84 (0.82–0.86)
r4s2	0.71 (0.69–0.74)	0.73 (0.70–0.76)	**0.76** (0.74–0.78)	0.67 (0.64–0.69)	0.73 (0.71–0.76)
r4s1	0.61 (0.59–0.63)	0.63 (0.60–0.65)	**0.65** (0.63–0.68)	0.58 (0.56–0.61)	0.63 (0.61–0.66)
r3s3	0.79 (0.77–0.82)	0.81 (0.78–0.84)	**0.84** (0.82–0.86)	0.75 (0.72–0.77)	0.81 (0.79–0.83)
r3s2	0.70 (0.67–0.72)	0.72 (0.69–0.74)	**0.74** (0.72–0.76)	0.65 (0.62–0.67)	0.70 (0.68–0.73)
r3s1	0.60 (0.57–0.62)	0.61 (0.59–0.64)	**0.64** (0.62–0.66)	0.57 (0.55–0.60)	0.61 (0.59–0.63)
r2s3	0.77 (0.74–0.79)	0.79 (0.76–0.81)	**0.82** (0.80–0.83)	0.72 (0.69–0.74)	0.74 (0.72–0.76)
r2s2	0.67 (0.65–0.69)	0.69 (0.66–0.72)	**0.72** (0.69–0.74)	0.62 (0.60–0.65)	0.64 (0.62–0.67)
r2s1	0.57 (0.55–0.59)	0.59 (0.56–0.62)	**0.62** (0.59–0.64)	0.56 (0.53–0.58)	0.57 (0.54–0.59)

**Table 2:** The number of instances that the null hypothesis (no difference between two tests) was rejected out of 1000 bootstraps (at a *P* < 0.05 level). In the column headings, 1 = GLR1, 2 = GLR2, 3 = GLR3, 4 = VS and 5 = *t*-test.

Data condition	Differences between AROC at *P* < 0.05 level									
	1,2	1,3	1,4	1,5	2,3	2,4	2,5	3,4	3,5	4,5
r8s3	15	949	946	952	993	925	956	1000	975	1000
r8s2	60	995	935	964	973	1000	64	1000	51	1000
r8s1	52	925	991	965	977	999	39	1000	16	1000
r6s3	47	941	951	952	979	928	970	1000	47	1000
r6s2	50	988	983	966	976	1000	17	1000	994	1000
r6s1	33	958	953	955	979	995	58	1000	964	1000
r4s3	36	999	966	991	991	995	25	1000	973	1000
r4s2	36	983	978	21	996	996	66	1000	954	1000
r4s1	32	988	994	64	956	946	18	1000	996	984
r3s3	48	995	992	40	988	996	66	1000	985	1000
r3s2	39	988	975	28	975	996	17	1000	999	990
r3s1	37	956	999	35	957	966	65	1000	910	983
r2s3	57	958	976	46	976	1000	929	1000	1000	957
r2s2	34	984	971	49	969	996	961	1000	1000	35
r2s1	15	977	977	23	996	995	28	994	993	12
